# The Burden of HIV, Hepatitis B and Hepatitis C by Armed Conflict Setting: The Nigeria AIDS Indicator and Impact Survey, 2018

**DOI:** 10.5334/aogh.3226

**Published:** 2021-06-25

**Authors:** Gambo G. Aliyu, Sani H. Aliyu, Akipu Ehoche, Deepa Dongarwar, Rafeek A. Yusuf, Muktar H. Aliyu, Hamisu M. Salihu

**Affiliations:** 1 National Agency for the Control of AIDS (NACA), Nigeria; 2 Addenbrooke’s Hospital, Cambridge, UK; 3 Maryland Global Initiatives, Abuja, Federal Capital Territory, Nigeria; 4 Baylor College of Medicine Center of Excellence in Health Equity, Training and Research, Houston, Texas, USA; 5 Department of Management, Policy, and Community Health, University of Texas Health Science Center at Houston, School of Public Health, Houston, Texas, USA; 6 Vanderbilt Institute for Global Health, Nashville, Tennessee, USA; 7 Addenbrooke’s Hospital, Cambridge, UK; Department of Family and Community Medicine, Baylor College of Medicine, Houston, Texas, USA

## Abstract

**Background:**

Against a background of security challenges, Nigeria conducted recently the largest population-based HIV survey in the world to ascertain the burden of the HIV disease in the country.

**Objective:**

We evaluated the main outcomes of the survey and the level of success using participation/response indicators.

**Methods:**

The survey was conducted from July–December 2018 by over 6,000 field staff across Nigeria in six consecutive webs, using two-stage cluster sampling. We estimated the prevalence of HIV, hepatitis B and hepatitis C in the entire country and by conflict zone status. Adjusted odds ratios (OR) and 95% confidence intervals (CI) from survey logistic regression models were used to compare the likelihood of test positivity for the three infections between zones.

**Findings:**

A total of 186,405 adults were interviewed from 97,250 households in 3,848 census enumeration areas. The overall HIV, hepatitis B and hepatitis C positivity rates were 1.55%, 7.63% and 1.73%, respectively. The prevalence of HIV, hepatitis B and C infection was significantly greater in conflict than non-conflict zones (HIV: 1.75% versus 1.0%; hepatitis B: 9.9% versus 7.3%; and hepatitis C: 3.2% versus 0.3%; p < 0.01 in all cases). Individuals living in conflict zones were about three times as likely to test positive for HIV (OR = 2.80, 95% CI = 2.08, 3.60) and nearly six times as likely to test positive for hepatitis C (OR = 5.90, 95% CI = 2.17, 16.67).

**Conclusion:**

Large population-based surveys are feasible, even in armed conflict settings. The burden of HIV, hepatitis B and hepatitis C was significantly higher in areas of conflict in Nigeria, highlighting the need for reinforced public health control measures in these settings in order to attain UNAIDS’ 95-95-95 targets of controlling the HIV epidemic in sub-Saharan Africa by 2030.

## Introduction

Population-based HIV Impact Assessment (PHIA) Surveys are national cross-sectional studies that quantify the impact of HIV programs in resource-constrained countries [1]. PHIA surveys, unlike the Demographic and Health Surveys and AIDS Indicator Surveys, directly measure HIV incidence, prevalence and viral load among persons living with HIV, expand the collection and management of HIV data, and enhance laboratory-based measurement of HIV status in survey countries [1]. PHIA surveys are often conducted in settings lacking strong HIV case-base surveillance systems, and their results can be used to guide HIV policy development and resource allocation in survey countries.

The conduct of research studies in armed conflict settings is associated with considerable strategic, methodological, applied, ethical, and policy challenges [2,3,4,5,6,7,8,9,10,11]. Nigeria is not immune to violent conflicts and faces similar problems related to conducting research in conflict settings [10,12,13]. In addition to security challenges from conflicts with Boko Haram and militia groups, Nigeria’s land mass, topography and population size pose additional challenges. In high conflict situations, the protection of human subjects extends beyond study participants to ensuring the safety and security of field data collectors, who in some situations may be more vulnerable than the research participants. The nature of armed conflicts in Nigeria necessitates the use of innovative approaches tailored to the conflict region to predict their occurrence and apply appropriate measures to avoid or avert them during the conduct of a nationwide study [14,15].

In 2018, Nigeria implemented its own PHIA survey, christened the Nigeria AIDS Indicator and Impact Survey (NAIIS). In this article, we describe the main outcomes of the NAIIS, which, to our knowledge, is the largest population-based HIV survey conducted so far in the world. Additionally, it is the only PHIA survey that integrated hepatitis B and hepatitis C burden estimation. Given the prevailing insecurity in some areas, we also describe innovative measures that were implemented to assure data quality and to ensure the safety and security of study participants and research staff.

## Methods

### Study design

The NAIIS project is a nationwide cross-sectional household survey conducted over a 22-week period from July 2018 to December 2018. The main goals of the project were to: 1) Ascertain the burden and distribution of HIV disease in Nigeria; 2) Assess the coverage and impact of HIV services at the population level; and 3) Measure HIV-related risk behaviors using a nationally representative sample of persons aged 0 to 64 years.

### Sampling

#### Target universe and Sampling Frame

The target population comprised all adults 15 to 64 years and children 0 to 14 years old within households across the 36 states and the Federal Capital Territory (FCT), Abuja in the six geo-political zones of Nigeria. The NAIIS utilized a national sample frame comprising all households in the enumeration areas (EAs) based on the 2006 National Population Census. The sampling frame consisted of 662,855 EAs, containing 28,900,478 households and 140,431,798 persons, with an average number of households and persons per EA of 44 and 212, respectively.

#### Sampling design

A two-stage cluster sampling design was employed, starting with EAs followed by households, similar to designs utilized by USAID’s Demographic and Health Surveys (DHS) [16], UNICEF’s Multiple Indicator Cluster Survey (MICS) [17], and other Nigerian national surveys [18].

*First-stage sampling (cluster selection)*: Within each of the 36 states in Nigeria, EAs were selected with Probability Proportional to Size (PPS) based on the 2006 census. Within states, the urban/rural distribution of selected EAs was proportional to their Census distribution.

*Second-stage sampling (household selection)*: Following first-stage sampling, the National Population Commission updated the cartographic materials and household listings of each selected EA. The updated list of households (with unique serial identifiers) served as the sampling frame for the selection of households in the second stage. Twenty-five households were selected in every EA. A uniform number of households per EA was utilized in order to standardize field operations. The selected households were visited without replacement or modifications. The expected number of missing households, by either refusal or absence, was considered in the sampling design by increasing the number of households surveyed in each EA to 28. During the listing, Global Positioning System was employed to facilitate household location and relocation of sampled households.

### Study Population and Sample Size Determination

This study was restricted to women and men aged 15–64 years living in residential households and visitors who slept in the household the night before the survey interview. During the individual interview, a subset of eligible participants, 18 to 54 years, was administered the network scale-up method (NSUM) module, comprising questions meant to estimate the size of key populations (men who have sex with men, female sex workers and their clients, and people who inject drugs).

The overall size and distribution of the sample was determined by analysis of existing estimates of national HIV incidence, sub-national HIV prevalence, and the number of HIV-positive cases needed to obtain prevalence estimates of viral load suppression among adults 15–64 years for each of the 36 states and the FCT. Overall sample sizes for the survey and blood draw populations were adjusted for survey non-response, variation in the household size by state, missing biomarker specimens, and sampling of children under 15 years for blood draw and testing.

### Study Measures

#### Variables

The individual adult questionnaire variables were categorized under 1) demographic characteristics; 2) reproductive history for women – antenatal care and PMTCT services uptake; 3) marriage and sexual activity, HIV-related risk behaviors; and 4) previous HIV testing experience, knowledge of HIV sero-status, and continuum (uptake) of HIV care services. The individual adolescent questionnaire (age 10 to 14 years) contained a subset of questions from the adult questionnaire, including demographic characteristics, sexual activity, HIV-related risk behaviors, exposure to HIV prevention programs, and knowledge of HIV sero-status.

Whole blood specimen testing included: 1) rapid HIV testing in home-based testing and counseling (HBTC); 2) CD4+ cell count testing for persons found to be HIV-positive following home-based testing and counseling, and 2% of those who tested HIV negative; and 3) Hepatitis B virus surface antigen (HBsAg) testing and Hepatitis C virus antibody testing for all HIV-positive individuals and approximately 5,303 randomly selected HIV-negative individuals age 15 to 64 years.

### Data Collection and Management

#### Survey data collection

Over 6000 field staff collected data for the survey from Nigeria’s six geo-political zones in six consecutive webs covering at least one state per web per zone. A total of 190 teams of 10 persons per team collected data across 36 states and the FCT over a period of 22 weeks. Field teams visited each selected household in sampled EAs, identified the head of the household, and introduced the purpose of the survey and the members of the team. A team consisted of a lead, a community guide, two interviewers, two counsellors, two laboratory staff and two drivers. Participants were interviewed in their households after obtaining relevant informed consent. Venous blood was collected using Ethylene Diamine Tetra-acetic Acid (EDTA)-containing vacuum tubes. Blood samples collected at the household were tested on the spot for HIV, CD4+ cell counts, HBsAg and HCV antibodies, as applicable.

#### Data entry, location and storage

All data from the household and individual questionnaires were entered directly onto mobile tablet devices using the Census and Survey Processing System (CSPro) pre-programmed with the questionnaire. SureMDM was used to secure, monitor and manage the tables devices, including loading survey software, encryption and password-protection, prevention of installation of non-survey software, and protection against security threats, including the ability to remotely lock and wipe devices in the event of theft or loss. Field CD4 data were pushed to the main survey server weekly. Results of specimens transported to the satellite and central laboratories were sent via a secure File Transfer Protocol server to the main survey server on a weekly basis. Survey and field laboratory data were synchronized daily to the main server. Data were backed up daily to a secured encrypted portable USB drive that was stored at a different location.

### Quality Assurance Measures

#### Planning, Implementation, and Evaluation

**Figure 1:**
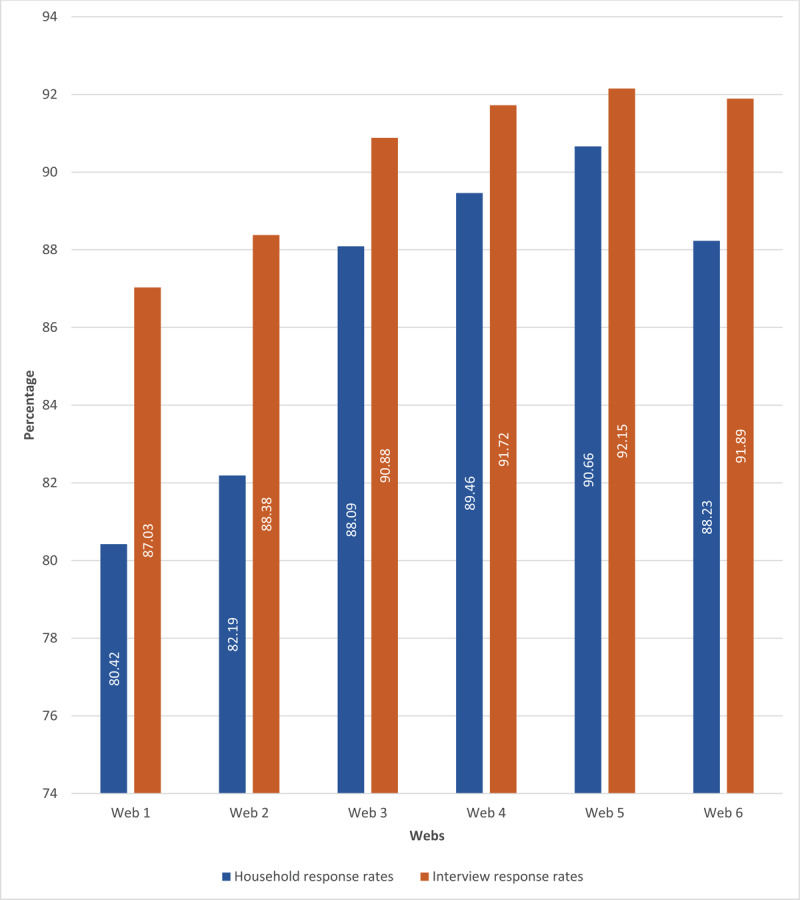
Household and interview response rates.

Novel quality assurance measures were incorporated at each phase of the survey. At the *planning* phase, culturally appropriate technology was used to ensure survey accuracy, confidentiality, integrity, and availability (***Figure 1***). This phase was comprised of the development of data systems architecture, activity information management systems (AIMS), laboratory data management systems, Census and Survey Processing System (CSPro)-Computer Assisted Personal Interview (CAPI) system, data monitoring center, and dash boards. A novel quality assurance measure introduced as part of the planning phase was survey piloting.

The *implementation* phase included a pilot run, which was conducted in each of the six geo-political zones. Problems in survey activities (e.g., household mapping and listing, satellite laboratory readiness, transport systems, staff safety measures, the CAPI system, unprotected access and arm-to-freezer time) were identified and resolved. An innovative feature of the implementation phase was the “web operational design,” whereby the survey was conducted in smaller dimensions (webs). The purpose of this design was to create a learning system that would minimize error over time. Field security operational measures were deployed to assure the physical security of survey staff and encompassed: careful and rigorous planning; pre-assessment security visits and engagements; intelligence gathering; engagements with community gate keepers, rulers, and stakeholders; and working with the Civilian Joint Task Force (CJTF) in high risk regions. Other security measures included real-time communication among teams with mobile applications such as WhatsApp; capacity building and training of field team members working in high risk areas on security awareness; security training of all drivers; reinforced/continuous communication on the need for security awareness; mapping and classification of survey areas based on threat levels; daily intelligence briefing by zonal leadership and security experts; and the creation of operational command centers.

In the *evaluation* phase, a robust and adaptable data architecture was created to allow for timely, effective, efficient, safe, and cost-effective data collection, transmission, monitoring, and reporting (Supplementary Figure 1). Proprietary devices such as CSPro Computer Assisted Personal Interviewing (CAPI), Encuesta, Abbott Pima, and Frontier LDMS were employed to capture data and eliminate sources of bias.

### Data Analysis Plan

We examined three indicators that captured the degree to which the NAIIS project was successful: the household response rate, the survey response rate and the blood draw acceptance rate. We defined the household response rate as: (Number of selected household units with completed interviews × 100) ÷ (Total number of selected household units). Survey response rate was defined as the number of eligible individuals in selected household units with completed interviews × 100 ÷ (total number of eligible individuals in selected household units). We defined the blood draw acceptance rate as the number of eligible individuals with blood draw × 100 ÷ (total number of eligible individuals with completed interviews). We also calculated the laboratory error rate as a quality assessment metric, defined as the proportion of rapid field HIV tests that was at variance from the confirmatory laboratory test results. We computed the HIV, Hepatitis B and Hepatitis C positivity rate as the proportion of tested individuals with a positive result for the specific test. Finally, using the Chi-square test, we compared the aforementioned survey outcomes between non-conflict and armed conflict zones for webs 5 and 6, the two webs during which the areas of armed conflict were entered and surveyed. A conflict zone was determined by the security sub-committee of the survey. The sub-committee comprised security experts in the country, and the designation of conflict zones was made based on the prevailing security situation at the time of the survey. Accordingly, the states described as conflict zones were: Benue, Borno, Plateau, Yobe and Zamfara. We employed survey logistic models to compare the likelihood of test positivity for the three infections between the two zones. We included the following covariates in the models: age, sex, place of residence (urban/rural), wealth quantile (categorized as lowest, second, third, fourth and highest stratum of household income) and web. All hypothesis tests were two-tailed with a type 1 error rate set at 5%, and all analyses were performed using SAS Version 9.4 (Cary, NC.).

### Ethical and Human Subjects Consideration

The protocol for the survey was approved by the Nigeria National Health Research Ethics Committee (NHREC) and the Institutional Review Boards at the CDC and the University of Maryland. Persons with disabilities were offered survey participation as long as they met the eligibility criteria. The survey did not include any prisoners or persons in jails/legal detention. The funders of the study played no role in drafting the manuscript.

## Results

A total of 186,405 adults were interviewed from 97,250 households in 3,848 census EAs. The overall household response rate (including households that could not be visited) was 86.50%, while the overall interview response rate was 90.34%. In ***Figure 1***, household response rates showed 9.71% relative increment from web 1 to web 6 (the final web) trending from 80.42% in web 1 to 88.23% in web 6 (p < 0.01, ***Figure 1***). Similarly, interview response rates rose from 87.03% in web 1 to 91.89% in web 6 corresponding to a 5.58% improvement (p < 0.01). The average blood draw acceptance rate over the six webs was 93.30%, with an increasing trend from 92.0% in web 1 to 93.90% in web 6 (p < 0.01) [data not shown].

**Figure 2:**
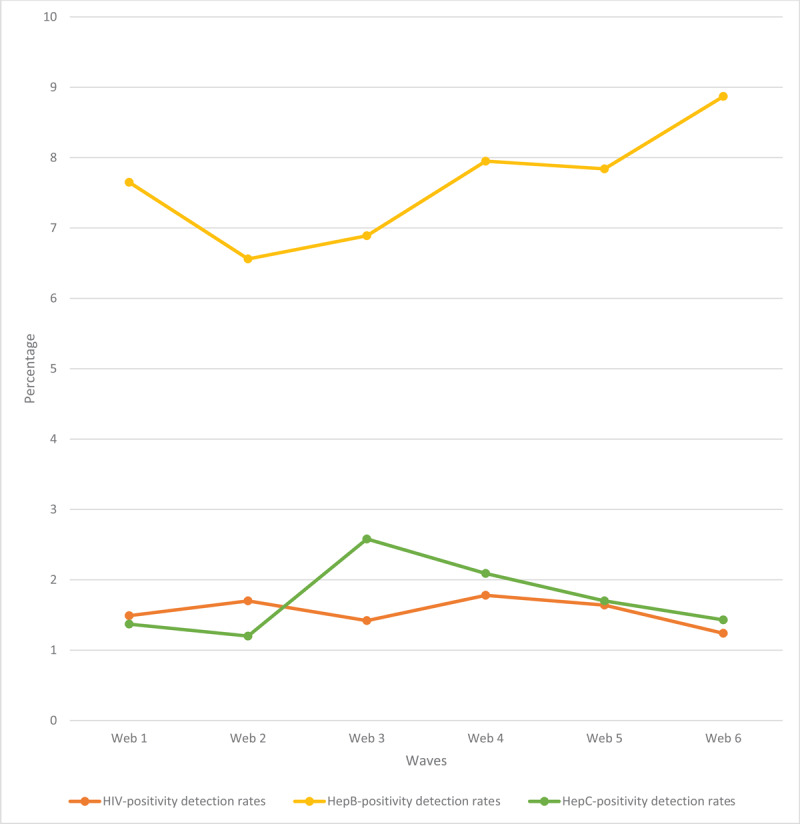
Positivity rates for HIV, Hepatitis B and Hepatitis C.

The overall HIV-positivity rate was 1.55% (***Figure 2***). The detection rate fluctuated over successive webs, with a peak HIV positivity rate in web 4 and the lowest rate in web 6. Hepatitis B positivity rate ranged from 6.56% (web 2) to 8.87% (web 6), yielding an average Hepatitis B positivity rate of 7.63% for the entire study. The rate of Hepatitis C detection ranged from 1.20% (web 2) to 2.58% (web 3) with an overall average of 1.73%. The lab test error rate yielded an overall average of 0.22% (range: 0.11% to 0.33%, ***Table 1***). The lab error rate was relatively stable over the first four webs (web 1 to web 4, average: 0.18%), but the error rate increased in webs 5 and 6 to an average of 0.30%. There was also a progressive decline in the number of days for data collection per web as the survey proceeded - the number of data collection days plunged from 30 days in web 1 to 13 days in web 6 (57% decline). The total number of rapid HIV field tests also dropped by 47%, from 40,250 in web 1 to 21,443 in web 6.

**Table 1:** Lab error rate in the Nigeria AIDS Indicator and Impact Survey (NAIIS) across webs.


WEB	*AVERAGE NUMBER OF DATA COLLECTION DAYS	*NUMBER OF RAPID HIV TESTS DONE	DISCREPANCIES BETWEEN FIELD AND LAB HIV TESTS	ERROR RATE (PROPORTION OF DISCREPANT TESTS)

1	30	40250	65	0.16

2	20	34298	55	0.16

3	19	40858	52	0.13

4	18	37924	40	0.11

5	17	32546	86	0.26

6	13	21443	71	0.33


* *P* for trend < 0.01.

***Table 2*** presents household and interview response rates by zone. Since the conflict zones were surveyed only in web 5 and web 6, the comparison could only be made for the two webs. In web 5, household response rate was 10.56% greater in non-conflict than conflict zones while interview response rates were similar. Blood draw acceptance rate was slightly higher (by 1.22%) in conflict zones. In web 6, the household response rate in both non-conflict and conflict zones declined although the fall was more pronounced in conflict zones (from 80.38% in web 5 to 57.13% in web 6, a 23.25% decline). The comparable level of decline in the non-conflict zone was only 2.22% (from 90.94% in web 5 to 88.72% in web 6). The interview response and blood draw acceptance rates were greater in conflict zones in web 6. The overall prevalence (both webs combined) of HIV, hepatitis B and C infections was significantly greater in conflict than non-conflict zones (HIV = 1.75% versus 1.00%; hepatitis B = 9.91% versus 7.30%; and hepatitis C = 3.22% versus 0.30%; p < 0.01 in all cases). Data comparing laboratory error rates classified by zones were, however, not available.

**Table 2:** Comparison of survey outcome rates between conflict and non-conflict zones, NAIIS 2018.


VARIABLE	WEB 5				P-VALUE	WEB 6				P-VALUE
	
	CONFLICT ZONE		NON-CONFLICT ZONE			CONFLICT ZONE		NON-CONFLICT ZONE		
			
	N	%	N	%		N	%	N	%	

Household response rate	4984	80.38	10647	90.94	<.0001	7504	57.13	5880	88.72	<.0001

Interview response rate	10172	92.31	21549	92.08	0.4700	10799	92.56	9535	91.13	0.0002

Blood draw acceptance rate	9390	94.42	19842	93.20	<.0001	9996	95.00	8689	92.61	<.0001

HIV Positivity rate	10413	2.54	22009	0.88	<.0001	11349	1.03	10031	1.13	0.4990

Hep B positivity rate	626	10.38	1020	6.27	0.0026	647	9.43	684	8.33	0.4825

Hep C positivity rate	626	3.51	1020	0.59	<.0001	647	2.94	0	0.00	<.0001**


** Fisher’s exact test used instead of Chi-square test. N = the total number for that row and specified zone.

Adjusted odds ratios for the likelihood of HIV, hepatitis B and Hepatitis C positivity in conflict versus non-conflict zones as well as socio-demographic predictors are summarized in ***Table 3***. Individuals living in conflict zones were about three times as likely to test positive for HIV (OR = 2.80, 95%CI: 2.08, 3.60), and six times as likely to test positive for hepatitis C (OR = 5.90, 95%CI: 2.17, 16.67). The adjusted odds for testing positive for hepatitis B were similar for conflict and non-conflict zones. Differences in risk distribution for the three infectious diseases was apparent across the selected socio-demographic and survey factors. Adult females exhibited a 43% lower likelihood of testing positive for HIV than their male peers (OR = 0.57, 95%CI: 0.48, 0.68), but were about twice as likely to test positive for hepatitis B (OR = 1.85, 95%CI: 1.27, 2.70). No sex-based differences in hepatitis C positivity were observed. Rural dwellers were less likely to test positive for HIV than their urban-dwelling counterparts (OR = 0.72, 95%CI: 0.53, 0.98) but this difference was not observed for hepatitis B and C. In general, the likelihood of testing HIV-positive was lower in persons belonging to the lower wealth quintiles. In contrast, in the case of hepatitis C, individuals in the lower three wealth quartiles had substantially higher adjusted odds of having hepatitis C compared to those in the highest strata. No association was found between wealth strata and hepatitis B status. A lower likelihood of HIV and hepatitis C positivity was observed among younger adults compared to persons aged 55–64 years. The opposite finding was noted for hepatitis B, as the adjusted odds of testing positive for Hepatitis B surface antigen increased with decreasing age.

**Table 3:** Association between survey respondents’ characteristics (exposures) and HIV positivity, Hepatitis B positivity, Hepatitis C positivity (outcomes).


CHARACTERISTICS	HIV POSITIVITY	HEPATITIS-B POSITIVITY	HEPATITIS-C POSITIVITY
		
	OR(95% CI)	OR(95% CI)	OR(95% CI)

**Conflict zone**			

No	Reference	Reference	Reference

Yes	2.80 (2.08,3.60)	1.10 (0.72,1.64)	5.90 (2.17,16.67)

**Gender**			

Male	Reference	Reference	Reference

Female	0.57 (0.48,0.68)	1.85 (1.27,2.70)	1.02 (0.44,2.36)

**Place of Residence**			

Urban	Reference	Reference	Reference

Rural	0.72 (0.53,0.98)	1.20 (0.80,1.79)	0.38 (0.09,1.69)

**Wealth Quintile**			

Lowest	0.39 (0.25,0.62)	1.42 (0.67,3.04)	13.0 (1.19,141.75)

Second	0.60 (0.39,0.91)	1.11 (0.54,2.28)	21.01 (2.51,175.67)

Third	0.65 (0.45,0.94)	0.94 (0.45,1.96)	19.58 (2.55,150.47)

Fourth	0.89 (0.66,1.21)	1.57 (0.8,3.09)	0.17 (0.07,0.43)

Highest	Reference	Reference	Reference

**Age Category**			

15-24 years	0.20 (0.13,0.30)	4.11 (1.77,9.5)	0.09 (0.02,0.36)

25-34 years	0.70 (0.5,0.0.98)	4.61 (1.98,10.72)	0.22 (0.06,0.74)

35-44 years	1.32 (0.96,1.82)	3.48 (1.49,8.11)	0.32 (0.10,1.10)

45-54 years	1.19 (0.86,1.65)	2.51 (0.97,6.53)	0.96 (0.37,2.47)

55-64 years	Reference	Reference	Reference

**Web**			

Web 5	Reference	Reference	Reference

Web 6	0.55 (0.42,0.71)	1.23 (0.83,1.84)	0.74 (0.34,1.61)


OR = adjusted odds ratio; CI = Confidence Interval.

## Discussion

Prior to the NAIIS project, Nigeria was using a combination of periodic surveys to obtain routinely data to monitor HIV epidemic trends. Examples of such surveys include the Antenatal Clinic (ANC) sentinel survey [19], NARHS [20], Integrated Biological and Behavioral Surveillance Survey (IBBSS) [21], etc. These survey approaches may not accurately capture HIV metrics that could be employed reliably to ascertain the distribution of HIV in Nigeria, to assess the coverage and impact of HIV services on the population level, and to measure HIV-related risk behaviors among all persons. As a Population-based HIV Impact Assessment (PHIA), the NAIIS study is therefore, timely, and responsive to a pressing necessity for a more accurate ascertainment of HIV disease burden and service needs of people living with HIV/AIDS.

In this study, the overall household response rate was 86.51% and the overall interview response rate was 90.34%. The interview response rates for HIV-related national population studies in other African countries range from 75.4% to 99.7% [22]. Similarly, the overall blood draw (for HIV testing) acceptance rate in our survey was 93.27%, which favorably compares with the 65.5% to 96.3% reported for sub-Saharan Africa [22]. However, the sample size in the NAIIS project was by far the largest of any HIV study conducted on the African continent [22,23], a perspective that is important in interpreting the impressive overall interview response rate of 90.34% and blood draw acceptance rate of 93.27% in the NAIIS project. In addition, the implementation of the NAIIS project was challenging, given the prevalent security threats posed by armed conflicts in certain areas of the country. Therefore, the overall household, interview and blood draw response rates reported in this study are indicative of a high quality and reliable population survey taking into consideration the contextual challenges.

The lab error rate for field HIV tests showed an uptick in the last two webs (web 4 and web 6) and this finding coincided with the tail end of the survey (November and December 2018). This could be caused by the rush and pressure to have the survey concluded within the time frame as the NAIIS project neared its end. Fortunately, a well-built quality assurance process for field tests comprising layers of confirmatory tests captured these errors, a testimony that the quality assurance infrastructure installed in the NAIIS study actually worked to ensure integrity and reliability of survey findings.

The NAIIS project revealed the considerable progress made within the previous two decades in reducing the burden of HIV in Nigeria. Compared with nearly two decades ago, the prevalence of HIV dropped by about 300% (from 5.8% in 2001 to 1.55 in 2018) [24]. Further, when compared to about a decade ago, HIV prevalence plummeted by about 200% (from 4.6% in 2008 to 1.55% in 2018) [24]. The decline in HIV prevalence likely reflects the impact of HIV prevention efforts over the same time period.

We found a hepatitis B surface antigen positivity rate of 7.63% in this study. Well-conducted and recent sero-prevalence studies performed outside the NAIIS framework placed the hepatitis B antigen prevalence in Nigeria to be between 5.0% to 13.0%, with an average of 9.9% [25,26,27,28]. The hepatitis B antigen prevalence we observed in the NAIIS study is in agreement with these reports and confirms the persistence and high endemicity of the infection in Nigeria, despite the availability of an effective vaccine. We also detected antibodies to the hepatitis C virus in 1.73% of the study population. Recent studies conducted in various parts of Nigeria reported hepatitis C sero-prevalence rates that ranged from 0.5% to 3.0%, with an average of 1.73% [28,29,30,31]. This perfect concordance is again evidence of the high quality and reliability of the NAIIS study. As one of the 194 member states of the World Health Organization, Nigeria endorsed in 2016 the global initiative for the eradication of hepatitis B and hepatitis C infections by the year 2030 [32]. Our findings suggest that hepatitis B and C infections still remain a public health threat in Nigeria.

A unique feature of the NAIIS project is its implementation in the form of webs, the purpose of which was to develop a learning system that minimized error over time, and to circumvent the security challenges posed in conflict settings within the country. The NAIIS was conducted in six consecutive uninterrupted webs and completed within a period of six months in all non-conflict zones. However, in conflict zones, the survey was implemented only in the last two webs for tactical reasons. First, by acquiring experience regarding unexpected challenges in the more secure areas, research staff and survey leadership would be better prepared to implement the survey in areas of conflict. Our results indicate that household response rates were generally lower in conflict than non-conflict zones, a finding that was not surprising. However, the interview response rate was similar in web 5 but slightly higher in conflict zones in web 6 while the blood draw acceptance rate was greater in conflict zones for both webs. These indices support the feasibility of conducting large-scale surveys in conflict settings provided adequate security measures to protect the lives of research personnel are put in place.

We recognize the potential generalizability limitations of the data from conflict zones. Previous studies have criticized the quality and reliability of surveys in conflict areas in that scientific sampling is hampered by the changing dynamics of the population structure, hence, the difficulty in obtaining a representative sample in those areas [33]. Our findings in this regard need therefore, to be interpreted with such shortcomings in mind. Another potential limitation in the study is our inability to incorporate reliable metrics in our analysis that could take into account the impact of seasonal weather variations on household and interview response rates, and how that could have influenced some of the differences noted between conflict versus non-conflict zones.

## Conclusion

In summary, we describe the major findings of a large population survey conducted in Nigeria. Our results of high household, interview response and blood draw acceptance rates show that large population-based surveys are feasible, even in armed conflict areas. The higher prevalence of HIV, hepatitis B and hepatitis C in zones of conflict in Nigeria highlight the need for robust public health control measures targeting these settings.

## Additional File

The additional file for this article can be found as follows:

10.5334/aogh.3226.s1 Supplementary Figure 1. Flow diagram of Nigeria AIDS Indicator and Impact Survey (NAIIS) Data Architecture.
