# Supplementary material for: The Burden of HIV, Hepatitis B and Hepatitis C by Armed Conflict Setting: The Nigeria AIDS Indicator and Impact Survey, 2018
Source: Ann Glob Health. 2021 Jun 25;87(1):53. doi: 10.5334/aogh.3226 (PMC8231456; doi:10.5334/aogh.3226)
Supplement: Supplementary Figure 1. — Flow diagram of Nigeria AIDS Indicator and Impact Survey (NAIIS) Data Architecture. [file agh-87-1-3226-s1.pdf]

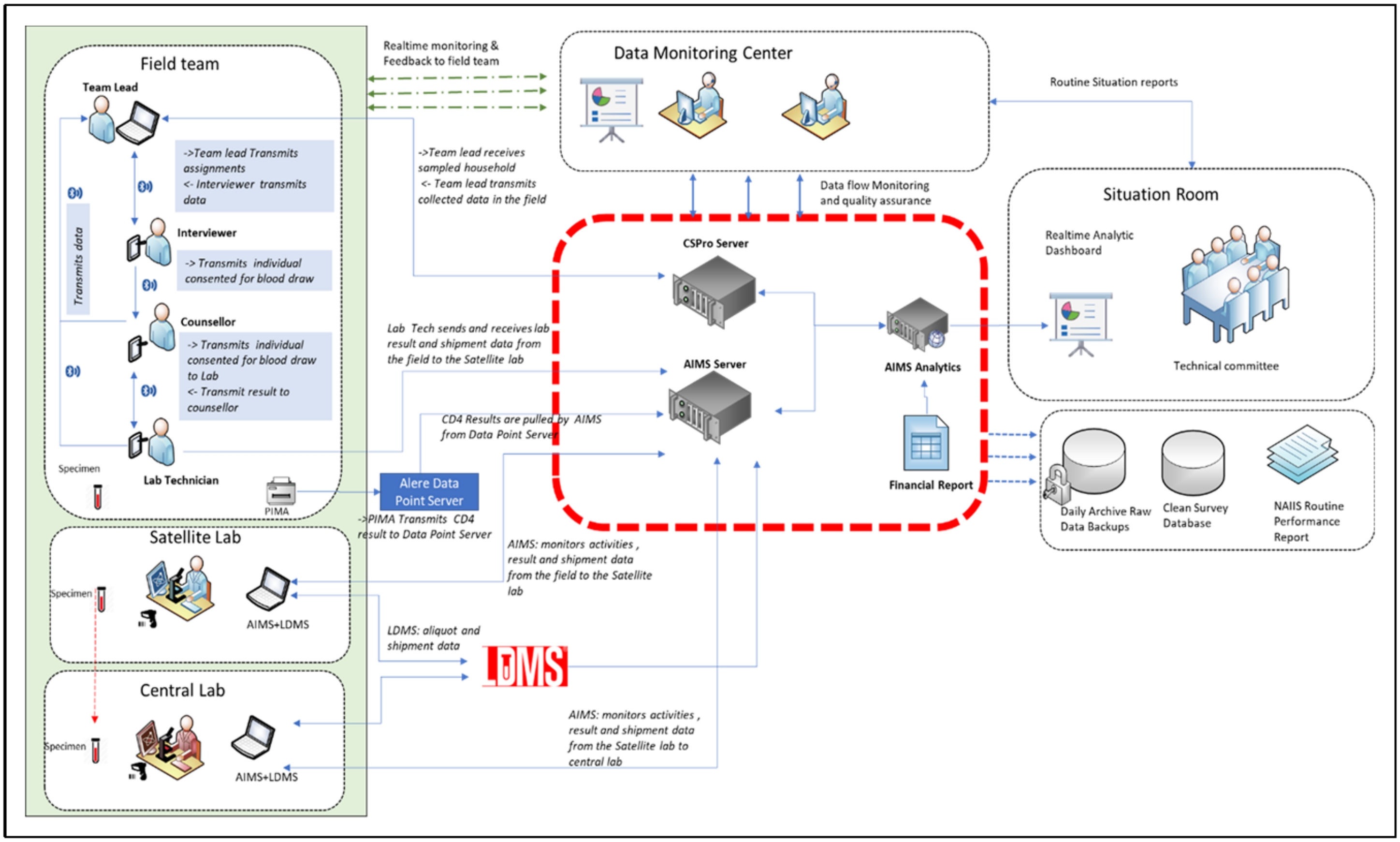

Supplementary Figure 1: Flow diagram of Nigeria AIDS Indicator and Impact Survey (NAIIS) Data Architecture.
